# Reversal Effect of Oxypeucedanin on P-glycoprotein-mediated Drug Transport

**DOI:** 10.3390/molecules23081841

**Published:** 2018-07-24

**Authors:** Wei Dong, Zhen-Gen Liao, Guo-Wei Zhao, Xue-Jing Guan, Jing Zhang, Xin-Li Liang, Ming Yang

**Affiliations:** Key Laboratory of Modern Preparation of TCM, Ministry of Education, Jiangxi University of Traditional Chinese Medicine, Nanchang 330004, China; sober96@foxmail.com (W.D.); lyzlyg@163.com (Z.-G.L.); weiweihaoyunqi@163.com (G.-W.Z.); gaojie.murong@outlook.com (X.-J.G.); evens_zhang@163.com (J.Z.); yangming16@126.com (M.Y.)

**Keywords:** oxypeucedanin (OPD), reversal effect, P-glycoprotein (P-gp), transmembrane transport

## Abstract

P-glycoprotein affects the transport of numerous drugs including chemotherapeutic drugs vincristine sulfate (VCR) and docetaxel (DTX), and is one of the main causes for multidrug resistance. Our previous studies have shown that oxypeucedanin (OPD) can enhance the intestinal transit of puerarin and VCR. However, the underlying mechanism is unclear. This study investigated the potential mechanism by which OPD improves P-gp-mediated drug transport. Molecular docking was performed to predict the binding force between OPD and P-gp and the contribution of OPD on P-gp activity. We observed the effect of OPD on the transport of VCR in MDCK-MDR1 cell monolayer and also measured the plasma pharmacokinetic parameters of DTX in the presence and absence of OPD by LC-MS/MS. Moreover, we further investigated the reversal mechanism of OPD on P-gp-mediated drug transport by determining the intracellular accumulation of Rhodamine-123 (Rh123) and P-gp ATPase activity as well as protein expression and mRNA level of P-gp. Our molecular docking results revealed that the binding force between OPD and P-gp was much lower than that between P-gp and verapamil (a P-gp substrate). The transport study in vitro indicated that OPD increased the flux of VCR across MDCK-MDR1 cell monolayer. The in vivo pharmacokinetic parameters data showed OPD increased the absorption of DTX. OPD activated P-gp ATPase activity and enhanced intracellular accumulation of Rh123 in MDCK-MDR1 cells. Western blotting and qRT-PCR outcomes indicated that OPD suppressed P-gp protein expression as well as downregulated P-gp mRNA level. Thus, OPD reverse P-gp-mediated drug transport via inhibition of P-gp activity and P-gp protein expression as well as downregulation of P-gp mRNA level. Our results suggest that OPD could reverse P-gp-mediated drug resistance in tumor cells.

## 1. Introduction

Multidrug resistance (MDR) has become a major cause of chemotherapy failure [1]. Many chemotherapeutic agents that are widely used for cancer treatment have been reported to cause multidrug resistance. For example, taxanes, vinca alkaloids, paeoniflorin and doxorubin, etc. [2]. Recent studies have revealed that overexpression of P-glycoprotein (P-gp) is the primary mechanism leading to the occurrence of drug efflux multidrug resistance [3]. P-gp, a 170-kDa energy-dependent transmembrane glycoprotein transporter encoded by human MDR1 gene, is widely expressed in the epithelial cells of normal tissues including intestine, liver, kidney, blood-brain barrier and placental barrier [4] and affects the absorption, distribution, metabolism and excretion of drugs. P-gp is also overexpressed in cancer cells and it can transport chemotherapeutic drugs out of the cells using the energy released by ATP hydrolysis [5]. The reduction of chemotherapeutic drugs concentration in cancer cells will result in poor efficacy for cancer therapy [6]. Therefore, it is of great importance to develop effective and non-toxic P-gp inhibitors to reverse P-gp-mediated multidrug resistance and improve the bioavailability of oral anticancer drugs.

With the development of traditional Chinese medicine scientific research and its clinical use, researchers have found that numerous Chinese herbs and especially their active ingredients play important roles in reversing P-gp-mediated multidrug resistance and improve the therapeutic effect of chemotherapy. To date, hundreds of compounds extracted from more than 40 species of plants have been reported to reverse drug resistance due to inhibiting P-gp activity. Tetrandrine, one bis-benzylisoquinoline alkaloid derived from *Radix Stephania tetrandra* was reported to have MDR-reversal activity via modulation of P-gp [7]. Quercetin, a native flavonoid, was found to bind to the promoter sequence of MDR1 gene and inhibit MDR1 gene expression in MDR cells [8]. In addition, a number of coumarins such as cnidiadin, galbanic acid, farnesiferol A, etc. have been proved to inhibit P-gp activity [9,10]. Recently, more and more attention has focused on the active ingredients of traditional Chinese medicine to explore and develop potential inhibitors of P-gp.

*Angelica dahurica* is a well-known traditional Chinese medicine with efficacy in “expelling wind and dispelling exopathogens, removing dampness and arresting leucorrhea, eliminating swelling and draining pus, and relieving pain”, according to traditional Chinese medicine theory. Previous studies indicated that oxypeucedanin (OPD), one of the main active ingredients of coumarins in *Angelica dahurica,* exhibits various pharmacological effects including anti-inflammatory, anti-depression, anti-tumor, protecting central nervous system, and also preventing skin diseases and AIDS, etc. [11,12,13,14,15]. Our previous study showed that *Angelica dahurica* suppressed P-gp-mediated drug efflux. Further study demonstrated that OPD enhanced the intestinal absorption of vincristine (VCR), which is affected by P-gp-mediated efflux. Our results suggest that OPD may reverse P-gp-mediated intestinal drug efflux. Thus, this present study was carried out to address the mechanism by which OPD inhibits P-gp-mediated drug efflux.

Mardin Darby canine kidney (MDCK) cells transfected with human MDR1 gene (MDCK-MDR1) has been established as an in vitro model for transport studies [16,17,18]. MDCK-MDR1 cells are characterized by high expression of P-gp and rapid differentiation [19]. In this study, we used MDCK-MDR1 cell model to observe the effect of OPD on the transport of VCR in MDCK-MDR1 cell monolayer. In addition, we also measured the concentration of docetaxel (DTX) in plasma of rats by LC-MS/MS to confirm whether OPD can affect the pharmacokinetic parameters of DTX. Moreover, we further investigated the reversal mechanism of OPD on P-gp-mediated drug transport by determining the intracellular accumulation of Rh123 and P-gp ATPase activity [20] as well as protein expression and mRNA level of P-gp. Our results demonstrated that OPD reversed P-gp-mediated drug transport via inhibition of P-gp activity and P-gp protein expression as well as downregulation of P-gp mRNA level. This study provides a research basis for the development of potential P-gp inhibitors and the improvement of clinical efficacy of chemotherapy drugs in future.

## 2. Results 

### 2.1. Molecular Docking Analysis

The docking of ligands into the active sites of proteins was determined by Libdock commercial software (Discovery Studio version 4.0, Accelrys, Scottsdale, AZ, USA), which can be used to calculate the hotspot map for the protein active site including polar and non-polar groups. The hotspot map was used to align the ligands that form favorable interactions and Libdock was used to perform energy minimizations for all ligand positions and ranks the ligands based on their scores [21,22,23]. The docking analysis results (Table 1) showed the interaction between ligand and P-gp. Numerous interactions were predicted between OPD and residues in the binding pocket, however, the LibDock Score of OPD is lower than that of verapamil. Our results indicated that OPD inhibited P-gp-mediated drug efflux may not be related to competitively binding with P-gp.

### 2.2. Effect of OPD on the Transport of VCR in MDCK-MDR1 Cell Monolayer

The effects of OPD and VCR on the viability of MDCK-MDR1 cells were observed by a MTT assay. Both OPD at the concentration of 3.82, 19.08, 76.30 μmol/L and VCR 264.46 μmol/L had no obvious cytotoxicity. Thus, we chose OPD (3.82, 19.08, 76.30 μmol/L) and VCR 264.46 μmol/L to investigate the role of OPD on the transport of VCR in MDCK-MDR1 cell monolayer. Compared to VCR alone group, the apparent permeability (*P_app_*) value (from apical to basolateral) of OPD 19.08, and 76.30 μmol/L combined with VCR group was significantly increased, and that of OPD 3.82 μmol/L combined with VCR group also had an obvious tendency to increase, respectively (Table 2). The result indicated that OPD increased the flux of VCR across MDCK-MDR1 cell monolayers.

### 2.3. Pharmacokinetic Experiments

**Figure 1:**
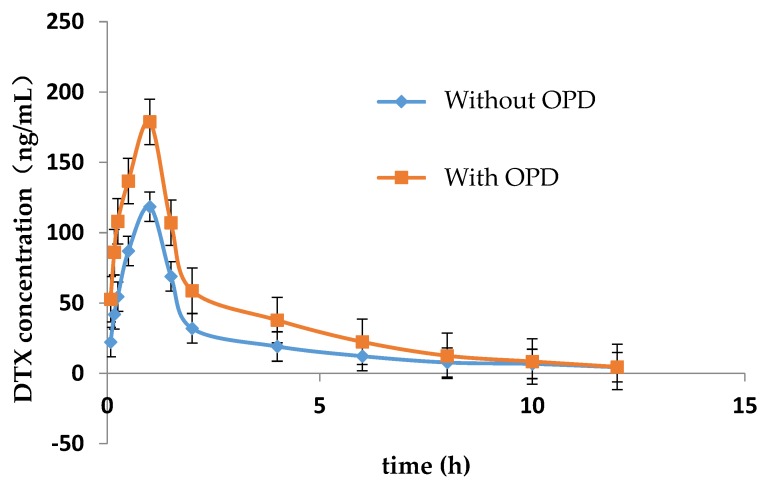
Mean plasma concentration-time curve of DTX in rats with or without OPD (*n* = 5).

The LC-MS/MS method for determining the concentration of DTX in plasma of rats was established in this study. Figure 1 showed the mean concentration-time curve of DTX in the presence and absence of OPD. The main pharmacokinetic parameters are summarized in Table 3. Compared with DTX alone group, *C*_max_, *AUC*_0–*t*_ and *AUC*_0–∞_ of DTX were significantly increased by 0.84, 1.21 and 1.49-fold, respectively, while *MRT*_0–∞_, *t*_1/2_, and *T*_max_ of DTX were significantly decreased in DTX combined with OPD group. No significant change were observed in *MRT*_0–*t*_ and *CL/F* of DTX in these two groups.

### 2.4. Effect of OPD on P-gp ATPase Activity

**Figure 2:**
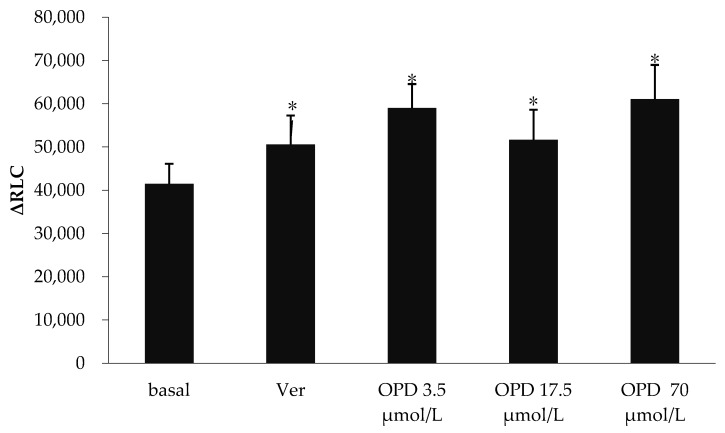
Effect of OPD modulating the activity of P-gp ATPase. Values are mean ± SD (*n* = 5). Differs from Basal group: * *p* < 0.05.

The effects of verapamil and OPD on P-gp ATPase activity were summarized in Figure 2. Verapamil is a substrate for transport by P-gp and has a stimulatory effect on P-gp ATPase activity. Compared to the basal P-gp ATPase activity, verapamil increased P-gp ATPase activity by 28.7%, while OPD resulted in a 0.55, 0.31 and 0.50-fold increase in P-gp ATPase activity at concentrations of 3.5, 17.5 and 70 μmol/L, respectively. Our data indicated that OPD could activate P-gp ATPase activity as well as verapamil although without obvious concentration-effect relationship.

### 2.5. Attenuation of OPD on Rh-123 Efflux

Rh-123, one of the specific P-gp substrates, has been used to determine P-gp activity in this study. The intracellular accumulation of Rh-123 is inversely proportional to P-gp activity. Our data summarized in Table 4 showed that verapamil significantly enhanced Rh-123 accumulation and inhibited P-gp transport activity in MDCK-MDR1 cells. OPD 3.5 μmol/L also significantly increased Rh-123 accumulation although OPD at concentrations of 17.5 and 70 μmol/L had only a tendency to increase the fluorescence intensity. Our result indicated that OPD could suppress P-gp transport activity in MDCK-MDR1 cells.

### 2.6. Inhibition of OPD on P-gp Protein Expression

**Figure 3:**
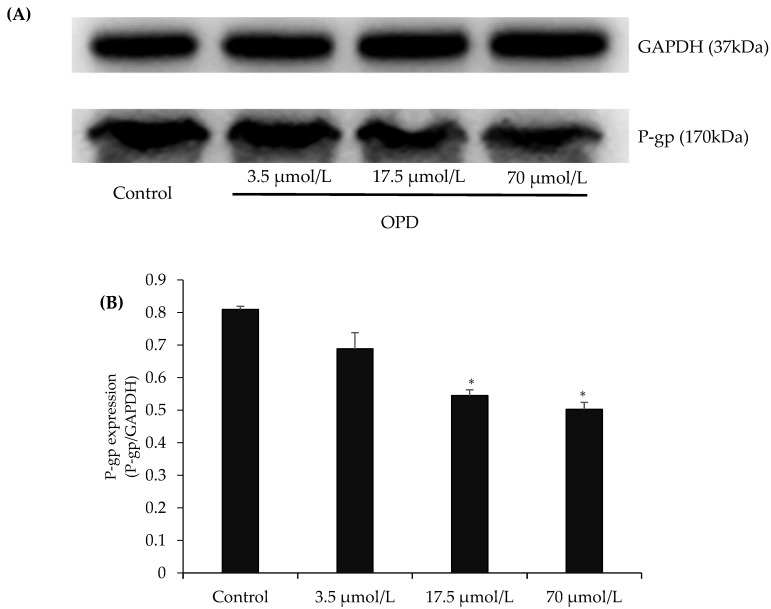
Effects of OPD on P-gp expression in MDCK-MDR1 cells: (**A**) representative P-gp expression by western blot on MDCK-MDR1 treated with different concentration of OPD; and (**B**) quantitative data of P-gp expression. Values are mean ± SD (*n* = 3). Differs from control group: * *p* < 0.05.

The representative western blots in Figure 3 showed that exposure of MDCK-MDR1 cells to OPD at concentrations of 17.5, and 70 μmol/L for 48 h resulted in an obvious reduction in P-gp protein expression in comparison with control group.

### 2.7. Downregulation of OPD on P-gp Gene Expression

**Figure 4:**
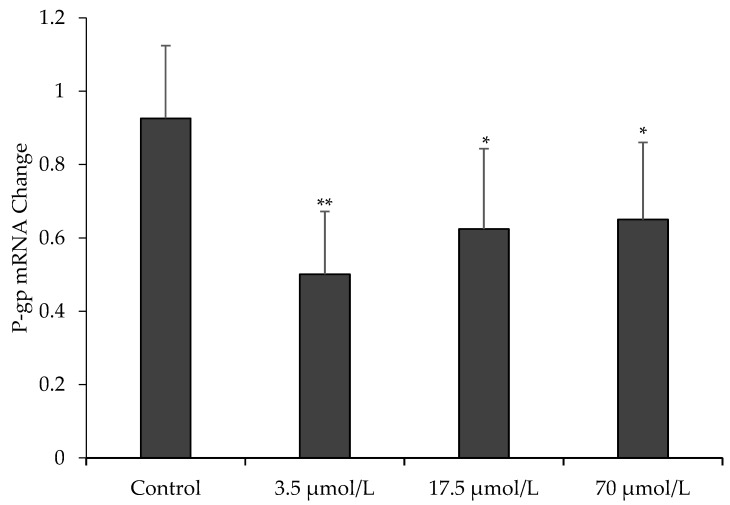
Effect of OPD on P-gp mRNA expression in MDCK-MDR1. Values are mean ± SD (*n* = 3). Differs from control group: * *p* < 0.05 ** *p* < 0.01.

To further assess the effect of OPD on P-gp function, we measured the mRNA expression of MDR1 in MDCK-MDR1 cells by quantitative real-time RT-PCR. Our data in Figure 4 showed that mRNA expression of MDR1 in MDCK-MDR1 cells treated with OPD at concentrations of 3.5, 17.5, and 70 μmol/L for 48 h were significantly downregulated compared to that of the untreated cells.

## 3. Discussion

P-gp-mediated multidrug resistance is one major obstacle of chemotherapy treatment in human cancers. P-gp protein has been considered as one of the most important targets for improving the efficacy of cancer chemotherapy [24]. Thus, many P-gp inhibitor candidates have been developed to reduce chemotherapy drugs efflux and thus enhance intracellular concentrations to overcome multidrug resistance and kill cancer cells. However, few of them can pass the clinical trials due to their severe side effects. In recent years, scientific researchers have spent huge efforts to search and screen P-gp inhibitor candidates from traditional Chinese medicine due to their non- or low-toxicity and safety. Numerous novel compounds derived from traditional Chinese herbs have been proved to have potential inhibitory effect of P-gp-mediated multidrug resistance [25,26].

In this study, for the first time, we assessed the role of OPD, an active coumarin compound in *Angelica dahurica*, on P-gp-mediated drug transport. Our data demonstrated that OPD could promote P-gp-mediated drug absorption as evidenced by enhanced transepithelial flux of VCR across MDCK-MDR1 cell monolayers as well as increased pharmacokinetic parameters of DTX mainly including the area under the plasma concentration–time curve (*AUC*_0–∞_) and the maximum plasma concentration (*C*_max_) in rats. Our results indicated that OPD enhanced the absorption of chemotherapy drugs in vitro and in vivo, the latter might be due to inhibition of P-gp-mediated DTX efflux in the intestinal absorption site. Our in vivo results also suggest there is a potential pharmacokinetic interaction between OPD and chemotherapy drugs.

Our molecular docking analysis preliminarily indicated that the mechanism that OPD inhibited P-gp-mediated drug efflux was different from verapamil and might not be related to competitively binding with P-gp. To understand potential mechanism, we focused on the measurements of P-gp ATPase activity and Rh-123 efflux. Increased P-gp ATPase activity and enhanced intracellular accumulation of Rh-123 result in decreased drug efflux. Our data indicated OPD at concentrations of 3.5, 17.5 and 70 μmol/L could activate P-gp ATPase activity although without an obvious concentration-effect relationship. Moreover, the activation effect is more potent than that of P-gp substrate verapamil. In addition, OPD 3.5 μmol/L significantly increased Rh-123 accumulation and OPD 70 μmol/L also had an obvious tendency to increase the fluorescence intensity. Thus, the reversal of OPD on P-gp-mediated drug efflux is due to inhibition of P-gp ATPase activity.

P-gp protein expression and MDR1 gene regulation are related to the occurance of multidrug resistance [27,28]. To fully explore the reversal mechanism of OPD on P-gp-mediated drug efflux, we further measured protein expression and mRNA level of P-gp after MDCK-MDR1 cells exposed to OPD treatment for 48 h, respectively. Our results demonstrated that OPD obviously suppressed P-gp protein expression and also significantly downregulated MDR1 mRNA level.

In conclusion, the reversal of OPD on P-gp-mediated drug transport is associated with inhibition of P-gp activity, and suppression of P-gp protein expression as well as downregulation of P-gp mRNA level. OPD is a substrate and potent P-gp inhibitor. It may have the potential to combine with chemotherapy drugs, thus resulting in attenuation of multidrug resistance and improvement of clinical efficacy for cancer therapy.

## 4. Materials and Methods

### 4.1. Materials

Oxypeucedanin (OPD) was purchased from the National Institutes for Food and Drug Control (Beijing, China). Vincristine sulfate (VCR) was provided by the National Pharmaceutical Engineering Center for Solid Preparation in Chinese Herbal Medicine, (NPEC, Nanchang, China). Docetaxel (DTX) was obtained from Xi’an Hao-Xuan Bio-Tech Co., Ltd. (Xi’an, China) Verapamil and rhodamine-123 (Rh-123) were products of Sigma-Aldrich (St. Louis, MO, USA). Trizol RNA extraction kit was purchased from Ambion (Thermo Fisher Scientific, Beijing, China). The reverse transcription kit and P-gp-Glo™ assay systems kit were provided by Promega (Madison, WI, USA). Primary antibodies for mouse monoclonal anti-P-gp (MDR) and mouse monoclonal GAPDH were obtained from Sigma-Aldrich, Inc. and Shanghai Bioleaf Biotech Co., Ltd. (Shanghai, China), respectively.

### 4.2. MDCK-MDR1 Cell Culture

MDCK-MDR1 Cells were obtained from Shanghai Zhongya Institute of Biological Genes Institute (CinoAsia Co., Ltd., Shanghai, China). Cells were cultured as previously described [29,30]. In brief, cells were cultured in high glucose DMEM medium supplemented with 10% fetal bovine serum (FBS), 1% non-essential amino acids, 1% l-glutamine, and a penicillin-streptomycin double antibiotic solution. Cells were incubated at 37 °C with 5% CO_2_ and cell medium was changed every other day. Cells grown to 80% confluence were digested with 0.25% EDTA-trypsin and passaged upon, and ready for experiments.

### 4.3. Molecular Docking

The molecular docking study was performed as previously described [29,30]. The three-dimensional protein structure of P-gp (PDB code: 3G60) was searched from the Brookhaven Protein Data Bank (PDB, http://www.rcsb.org). The molecular structures for the ligands including OPD, VCR and paclitaxel were obtained from NCBI and their SDF file format was downloaded, respectively. Discovery Studio 4.0 (DS) was used to perform the docking of the compounds into the active sites of P-gp through the following operations including removing water and other hetero atoms, adding hydrogen followed by protonation, ionization, and energy minimization. The CHARMmforce field was applied for geometry optimization. The P-gp residues identified according to previous literatures [29,30,31] were used to define an active pocket. The ligand was docked at the defined active site of P-gp using Libdock, and the Libdock score was recorded for analysis, respectively.

### 4.4. Transport Studies of OPD in MDCK-MDR1 Cell Model

The effect of OPD on the transport of VCR in MDCK-MDR1 cell monolayer was observed according to the methods described previously [30]. Briefly, cell monolayers were pre-incubated in Hank’s buffered salt solution (HBSS, pH 7.4) for 30 min at 37 °C. Trans-epithelial electric resistance (TEER) values of the MDCK-MDR1 cell monolayer ranged from 500 to 800 Ω·cm^2^. All drugs were dissolved in DMSO and diluted in HBSS. The final concentration of DMSO is less than 1%. Cells were washed for 3 times by pre-warmed HBSS and treated by OPD at concentrations of 3.82, 19.08, 76.30 μmol/L in the presence and absence of VCR 264.46 μmol/L. 0.5 mL of drug solution was added to the apical (A) side and 1.5 mL of HBSS was added to the basolateral (B) side to measure A→B transport. After treatments, cells were incubated at 37 °C for 2 h. After 2 h, 500 µL aliquots were taken from the basal side, mixed with 500 µL methanol, vortexed 2 min and then centrifuged for 10 min at 16,000 rpm. The supernatants were collected to measure the concentrations of compounds by high performance liquid-chromatography/mass spectroscopy (LC-MS/MS). Apparent permeability (*P_app_*) values are calculated from the relationship:

$$\;P_{app}=\frac{dQ/dt}{A\times C_{0}}$$

where *dQ*/*dt* is the cumulative transport rate of the compound on the receiving side (µg/s), *A* is the surface area of the cell monolayer (cm^2^), and *C*_0_ is the initial concentration in the donor compartment (µg/mL).

### 4.5. Pharmacokinetic Parameters Measurements

The animal experiment in this study was performed in accordance with the National Institutes of Health Guide for the Care and Use of Laboratory Animals and was approved by the Animal Ethics Committee of Jiangxi University of Traditional Chinese Medicine (JZLLSC2018-0023). Ten 8-week-old male Sprague-Dawley rats were randomly divided into the following two groups (5 rats in each group). Group 1: rats were administered a single oral dose of 50 mg/kg of DTX dissolved in 0.5% CMC-Na. Group 2: rats received a single oral administration of 50 mg/kg DTX and 8 mg/kg OPD dissolved in 0.5% CMC-Na. Animals were fasted for 12 h prior to experiment, but continued to have free access to water during this time. After administration, 0.5 mL of blood was collected from retinal venous plexus into the heparinized centrifuge tube at the following time points: 0.083, 0.167, 0.25, 0.5, 0.75, 1, 1.5, 2, 3, 4, 6, 12 and 24 h, respectively. Blood samples were centrifuged for 10 min at approximately 4000 rpm. 100 µL of plasma was collected from the upper layer, and mixed with 20 µL of taxol internal standard solution for 30 s followed by 500 µL of methanol for 3 min, then centrifuged at 15,000 rpm for 10 min. The supernatant was separated and collected. 5 μL of the supernatant was injected into the LC-MS/MS system for detection and quantification.

Separation was performed on a ZORBAX Extend-C18 (2.1 × 100 mm, 3.5 µm, Agilent, Palo Alto, CA*,* USA). The column oven was maintained at 25 °C. The mobile phase was 0.1% acetic acid as mobile phase A and acetonitrile as mobile phase B. The liner gradient elution program was set according to preliminary tests: 70% B, 0–0.01 min; 70%–52% B, 0.01–1.00 min; 52% B, 1.00–3.00 min; 52%–30% B, 3.00–3.10 min; and 30% B, 3.10–5.00 min, with the flow rate kept at 0.4 mL/min.

### 4.6. P-gp ATPase Activity Assay

P-gp ATPase activity was measured using P-gp-Glo™ assay systems kit according to the manufacturer’s instructions, the method detail of which was also described in our previous study [29]. The P-gp-Glo™ assay system kit mainly contains recombinant human P-gp membranes, ATP detection substrate, ATP detection buffer., P-gp-Glo™ assay buffer, Mg-ATP, verapamil (a positive control substrate of P-gp), and sodium orthovanadate (Na_3_VO_4_, a P-gp ATPase inhibitor). This study was performed to observe the effect of OPD on P-gp ATPase activity and a total of 6 groups (*n* = 3 for each group) were included as follows: the untreated group, the Na_3_VO_4_ group, the verapamil 100 µmol/L group, and OPD at concentrations of 3.5, 17.5 and 70 μmol/L groups. Those treatments were added into the solution containing 25 mmol/L Mg-ATP and 1.25 mg/mL recombinant human P-gp membranes, respectively and incubated at 37 °C for 40 min. After 40 min, ATP detection reagent was added, respectively. The unconsumed ATP detection reagent reacted with luciferase at room temperature for 20 min and the chemiluminescence intensity was detected. The average relative light units (RLU) was calculated as follows:

RLU_Na3VO4_ − RLU_NT_ = ΔRLU_basal_ (1)

RLU_Na3VO4_ − RLU_TC_ = ΔRLU_TC_ (2)

ΔRLU_basal_, the decrease in luminescence of the untreated group (RLU_NT_) compared to the Na_3_VO_4_ group (RLU_Na3VO4_), represents the basal P-gp ATPase activity. ΔRLU_TC_, the decrease in luminescence of the test compound-treated group (RLU_TC_) compared to the Na_3_VO_4_ group (RLU_Na3VO4_), indicates the P-gp ATPase activity in the presence of the test compound. The decrease in luminescence reflect ATP consumption by P-gp and is proportional to P-gp activity.

### 4.7. Rhodamine-123 Accumulation

P-gp activity was determined by measuring intracellular accumulation of Rh-123 in MDCK-MDR1 cells as previously described with some modification [32]. Briefly, MDCK-MDR1 cells were washed with HBSS for 3 times, and then collected through digestion and centrifugation (1000 rpm, 3 min). Cells were resuspended in culture media at a concentration of 1 × 10^6^ cells/mL and inoculated into 10 mL Eppendorf tube, 1 mL/tube. 3.5, 17.5 and 70 μmol/L of OPD and 100 µmol/L of verapamil was added in each tube (1 mL/tube), respectively and incubated with cells at 37 °C for 1 h. After 1 h, cells were washed twice with cold PBS and then incubated with 500 µmol/L Rh-123 for 1 h, respectively. Cells were washed twice with cold HBSS solution to terminate transportation. Intracellular levels of Rh-123 were detected by FITC channel using MoFlo XDP flow cytometer (Ex = 466 nm; Em = 535 nm, Beckman Coulter, Fort Collins, CO, USA).

### 4.8. Western Blotting

MDCK-MDR1 cells treated with different concentrations of OPD (3.5, 17.5 and 70 μmol/L) for 48 h were subject to western blotting for determination of P-gp protein level. Cells were washed 3 times with PBS. Total protein was extracted and the protein concentrations were measured according to the manufacturer’s instructions. Cell protein were denatured at 70 °C for 10 min. Protein samples with equivalent amounts were loaded and separated by 10% SDS-polyacrylamide gel electrophoresis and transferred to a polyvinylidene difluoride membranes. Membranes were blocked in 5% powdered non-fat milk solution for 4 h at room temperature and incubated with anti-P-gp primary antibody (GADPH was used as a loading control) overnight at 4 °C followed by an goat anti-mouse IgG secondary antibody (1:5000 dilutions) for 4 h at room temperature. Membranes were incubated with an ECL chromogenic solution for 4 min in the dark. The target protein bands were detected and protein intensity was calculated by a gel imaging system (Beta 3, Bio-Rad, Irvine, CA, USA).

### 4.9. Quantitative Reverse Transcription PCR (RT-qPCR)

MDCK-MDR1 cells were cultured for 5 days and treated with different concentrations of OPD (3.5, 17.5 and 70 μmol/L) for 48 h to measure MDR1 gene expression level. The total celluar RNA was extracted by the RNA easy Total RNA mini kit and reverse transcribed to cDNA with reverse transcription kit according to the manufacturer’s protocol. PCR primer design and synthesis were performed by the primer design software provided by Applied Biosystems. Primer sequences for MDR1 and hRpIPIv-F (loading internal control) genes are as follows: human hRpIP1v-F forward, 5′-CCCTCATTCTGCACGACGAT-3′; human hRpIP1v-F reverse, 5′-GGCTCAACATTTACACCGGC-3′; human MDR1 forward, 5′-TGGGGCTGGACTTCCTCTCATGATGC-3′, human MDR1 reverse, 5′-GCAGCAACCAGCACCCCAGCACCAAT-3′. PCR amplification was performed using Power SYBR Green PCR Mix according to the manufacturer’s protocol (Life Technologies, Carlsbad, CA, USA).

### 4.10. Statistical Analysis

All data were presented as the mean ± SD. Data were analyzed using one-way ANOVA followed by Student-Newman-Keuls test for multiple comparisons to determine group differences. *p* < 0.05 was considered statistically significant.

## Figures and Tables

**Table 1:** The detailed binding modes of ligands with P-gp and the LibDock Socre.

Compounds	The Type of Interaction	The Key Amino Acids	LibDock Score
Verapamil	Hydrogen Bond Hydrophobic	GLY222 ALA225	132.634
OPD	Hydrogen Bond Hydrophobic	SER729 PHE728, PHE71, PHE953, PHE974	106.192

**Table 2:** Effects of OPD on transport of VCR in MDCK-MDR1 cell monolayers ($\bar{x}\pm s$, *n* = 3).

Group	*P_app_* (A→B) (×10^−6^ cm/s)
VCR (264.46 μmol/L)	0.37 ± 0.04
VCR (264.46 μmol/L) + OPD (3.82 μmol/L)	0.47 ± 0.07
VCR (264.46 μmol/L) + OPD (19.08 μmol/L)	0.50 ± 0.04 *
VCR (264.46 μmol/L) + OPD (76.30 μmol/L)	0.52 ± 0.04 *

*P_app_*, permeability; A, apical side; B, basolateral side. Differs from VCR (264.46 μmol/L): * *p* < 0.05.

**Table 3:** Change of pharmacokinetic parameters of DTX in rats with or without OPD ($\bar{x}\pm s$, *n* = 5).

Pharmacokinetic Parameters	Unit	DTX	DTX + OPD
*AUC*_0–*t*_	μg·h/L	276.20 ± 19.13	463.90 ± 47.29 **
*AUC*_0–∞_	μg·h/L	306.40 ± 21.15	482.10 ± 48.69 **
*MRT*_0–*t*_	h	2.96 ± 0.22	2.89 ± 0.22
*MRT*_0–∞_	h	4.29 ± 0.98	3.36 ± 0.38 *
*t*_1/2_	h	4.13 ± 1.08	2.64 ± 0.39 **
*CL/F*	L/h/kg	1.00 ± 0.00	1.00 ± 0.00
*T*_max_	min	163.80 ± 11.82	104.60 ± 11.68 **
*C*_max_	μg/L	118.40 ± 10.93	178.80 ± 8.81 **

Differs from DTX group: * *p* < 0.05, ** *p* < 0.01.

**Table 4:** Effect of OPD on the accumulation of Rh-123 ($\bar{x}\pm s$, *n* = 3).

Group	Fluorescence Intensity
Rh-123	226,201 ± 28,927
Verapamil + Rh-123	422,897 ± 37,088 **
OPD (3.5 μmol/L) + Rh-123	281,754 ± 19,159 *
OPD (17.5 μmol/L) + Rh-123	235,766 ± 31,090
OPD (70 μmol/L) + Rh-123	273,655 ± 60,007

Differs from Rh-123 group: * *p* < 0.05, ** *p* < 0.01.
